# Ecological recovery trajectories of coastal wetlands following oil-well abandonment in the Yellow River Delta

**DOI:** 10.3389/fpls.2026.1989517

**Published:** 2026-10-22

**Authors:** DanDan Rong, Min Song, Yongjia Han, Changzhi Yang, Wenjun Wei, Yan Lin, Duoqiang Zhai

**Affiliations:** 1 Shandong Yellow River Delta National Nature Reserve Management Committee, Shandong, China; 2 School of Environment, State Key Laboratory of Wetland Conservation and Restoration, Yellow River Estuary Wetland Ecosystem Observation and Research Station, Beijing Normal University, Beijing, China; 3 Yantai Blue Sky Construction Engineering Co., Ltd., Shandong, China; 4 Hydrological Center of Weifang, Shandong, China

**Keywords:** coastal wetlands, ecological restoration, heavy-metal risk, oilfield retirement, plant community reorganisation, Yellow River delta

## Abstract

**Introduction:**

Oil-well retirement ends production but may leave physical, chemical and ecological legacies in coastal wetlands.

**Methods:**

We assessed soil conditions, heavy-metal risk and vegetation across retirement stages in the Yellow River Delta, China, in 2021, using 15 oilfield-associated sites and six natural reference wetlands. Sites were classified as Active, Abandoned, Restored (infrastructure removed and restoration implemented) or Reference, with retired sites assigned to <3, 3–5 and 5–10 year classes.

**Results:**

Oilfield sites had lower mean vegetation cover than reference wetlands in June (50.29% versus 69.00%) and December (38.00% versus 56.23%), together with higher pH and generally lower salinity. Cd accounted for 54.3-65.7% of potential ecological risk, and its single-factor index exceeded one at three sites. Mean cover and richness at Restored sites increased across age classes, from 15.8% and 1.5 species at <3 years to 64.2% and 7.0 species at 5–10 years, whereas diversity peaked in the intermediate class. Cd index, pollution load index (PLI) and potential ecological risk index (RI) did not decline monotonically with retirement age. Restored sites had lower mean metal-risk indices than Abandoned sites at 5–10 years but higher means at 3–5 years. Soil pH was positively associated with cover and richness; Cd index and PLI showed no significant associations with vegetation metrics.

**Discussion:**

These descriptive chronosequence patterns indicate that vegetation reorganisation and chemical-risk attenuation do not follow a common trajectory, and that recovery cannot be inferred from retirement age alone. Wetland retirement management should combine site-specific contamination assessment with vegetation and habitat evaluation.

## 1 Introduction

Coastal wetlands occupy the dynamic interface between land and sea and provide disproportionately important services, including biodiversity support, carbon storage, shoreline protection and water purification (Zedler and Kercher, 2005; Barbier et al., 2011). Wetland persistence also depends on feedbacks between vegetation and geomorphic processes (Kirwan and Megonigal, 2013). Many petroleum provinces overlap spatially with deltas, estuaries and coastal plains. In these settings, well pads, roads, dikes, drainage channels and accidental releases can modify the geomorphic, hydrological, soil and biological processes that sustain wetlands. The resulting effects can persist after activity has ceased as ecological legacies (Cuddington, 2011).

Research on oilfield wetlands has largely examined ecological effects during active production, including contamination, vegetation degradation, habitat fragmentation and reduced hydrological connectivity. In the Yellow River Delta, oil wells and associated roads have contributed to landscape fragmentation (Bi et al., 2011), while more recent analyses have shown that oil infrastructure can reduce wetland connectivity and alter conservation priorities (Wu et al., 2023). Studies following major oil spills further demonstrate that plant regrowth and habitat recovery need not coincide: vegetation can recolonise surviving marsh surfaces even where erosion has permanently removed part of the marsh platform (Silliman et al., 2012). This distinction between plant regrowth and habitat recovery is also relevant when assessing wetlands after production cessation. Production cessation does not, however, remove the footprint of pads, roads, stations or dikes. Soil compaction, altered microtopography, hydrological barriers and residual contaminants may continue to constrain plant establishment and succession after production ends.

Ecological recovery is not a unidirectional change in a single indicator. It involves reorganisation of ecosystem components and, potentially, convergence towards a reference condition. Recovery pathways can differ with the type of stressor and the physical setting of coastal ecosystems (Borja et al., 2010). Restoration studies show that structural and functional recovery can be incomplete and uneven across ecosystem components (Moreno-Mateos et al., 2012, Moreno-Mateos et al., 2017). In coastal wetlands, plant establishment depends on interacting constraints imposed by elevation, inundation and sediment conditions (Davy et al., 2011). Rapid recovery of species richness can consequently coexist with persistent differences in community composition between restored and natural marshes (Mossman et al., 2012). A reduction in contamination risk may likewise not coincide with the re-establishment of characteristic vegetation. Spatial chronosequences offer a practical means of examining these contrasting recovery patterns across sites of different ages (Walker et al., 2010).

Heavy metals provide an important chemical dimension of this assessment because their environmental behaviour differs from the physical removal of oilfield infrastructure. Their mobility and availability depend on sorption, sediment redox conditions, salinity and interactions with organic matter (Du Laing et al., 2009). Wetland plants can also differ in their uptake, retention and release of metals (Weis and Weis, 2004). In the Yellow River Delta, trace-element distributions vary among vegetation types and seasons and are associated with soil properties and water-sediment dynamics (Bai et al., 2014; Zhao et al., 2021; Yu et al., 2022). Thus, a regional mean concentration may conceal local enrichment, while a lower pollution index does not by itself establish that plant communities have recovered. Joint assessment of site-level contamination and vegetation is needed to determine whether chemical and biological responses agree.

Post-retirement management may further influence recovery pathways. Abandonment, in which production stops but pads or facilities remain, differs fundamentally from infrastructure removal followed by ecological restoration. Retained infrastructure can preserve physical legacies after operational disturbance has ended. Conversely, removal may improve surface conditions and habitat continuity, but its ecological effect requires direct evaluation rather than assumption. A global synthesis found variable benefits of active restoration relative to the cessation of disturbance alone, highlighting the need to relate intervention outcomes to the obstacles limiting recovery at particular sites (Jones et al., 2018). A wetland meta-analysis likewise found that restoration outcomes varied with the degradation history, restoration actions and ecosystem type (Meli et al., 2014). For oilfield wetlands, an important question is therefore whether restored and abandoned sites of similar retirement ages differ consistently in both vegetation condition and contamination risk.

The Yellow River Delta provides a suitable setting in which to examine these questions. This young estuarine wetland complex has experienced long-term petroleum development associated with the Shengli Oilfield, alongside wetland loss and hydrological change (Zhang et al., 2016; Zhu et al., 2018). Previous restoration studies in the delta have linked vegetation development to freshwater supply, water depth and soil salinity, with vegetation attributes responding differently over restoration time (Cui et al., 2009; Wang et al., 2012). Recent mapping also documents spatial variation in vegetation composition and structure across delta mudflats, providing a regional context for interpreting differences among sites (Li et al., 2024). Active facilities, retired sites and relatively undisturbed wetlands occur within the same regional landscape, allowing these restoration questions to be examined specifically in relation to oilfield retirement.

We examined soil conditions, metal-risk indices and vegetation across Active, Abandoned, Restored and Reference wetlands, with retired sites grouped into <3, 3–5 and 5–10 year classes. The analysis addressed three questions: (i) whether metal-risk indicators decreased with retirement age; (ii) whether vegetation attributes became closer to reference conditions; and (iii) whether Abandoned and Restored sites differed at comparable ages. As a complementary analysis, we examined associations between soil conditions, contamination indices and vegetation to identify environmental correlates of the observed patterns. Together, these comparisons distinguish the cessation of production from the ecological condition of the wetland and assess whether chemical and biological components exhibit concordant recovery trajectories.

## 2 Materials and methods

### 2.1 Study area and monitoring design

The study was conducted in oilfield-associated coastal wetlands of the Yellow River Delta, Shandong Province, China (**Figure 1**). Bordering the Bohai Sea, the delta contains a mosaic of freshwater-influenced and saline wetlands shaped by river inputs, tidal processes and human modification (Zhang et al., 2016; Zhu et al., 2018). Typical wetland vegetation includes *Phragmites australis*, *Suaeda salsa* and *Tamarix chinensis*, whose distributions vary along local water and salinity gradients (Bai et al., 2014; Wang et al., 2012). Petroleum infrastructure associated with the Shengli Oilfield occurs within this wetland landscape.

**Figure 1:**
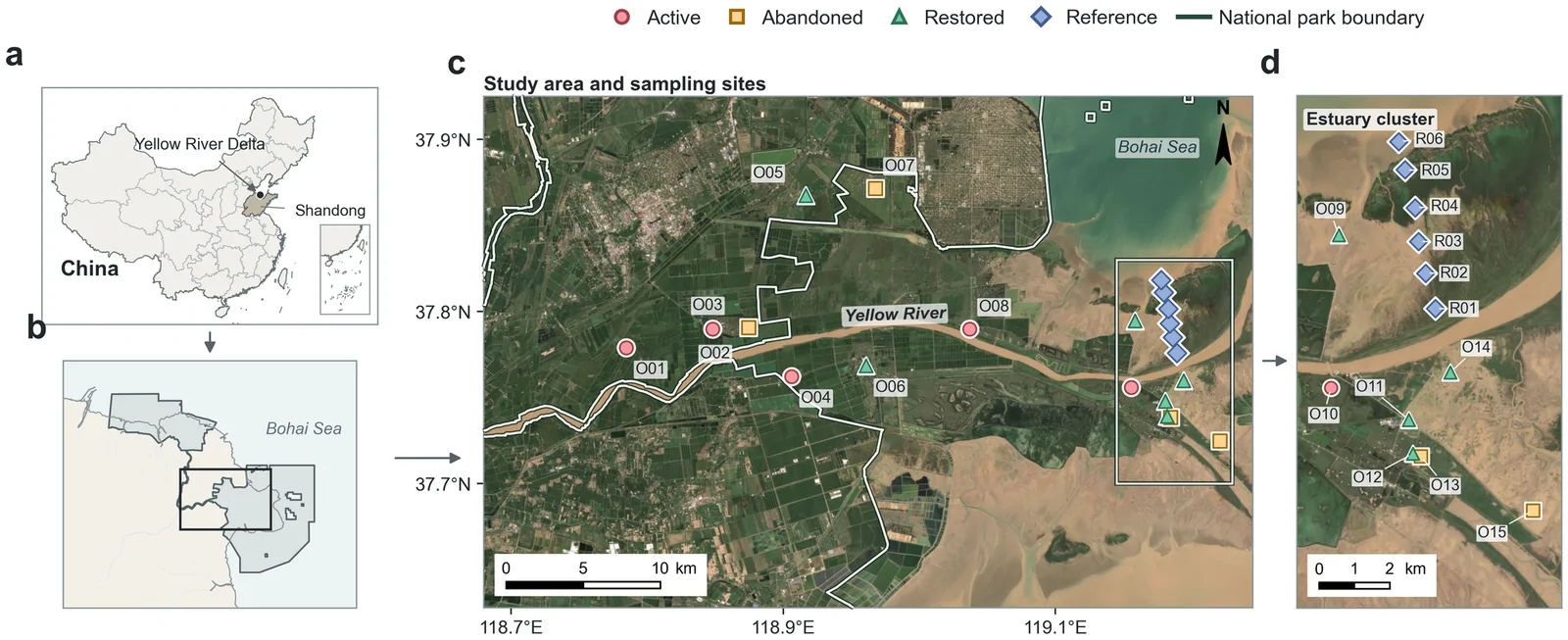
Study area, sampling sites and operational states of oilfield-associated wetlands in the Yellow River Delta. **(a)** Location of the Yellow River Delta within China. **(b)** Regional setting within Shandong Province. **(c)** Study area and sampling sites. **(d)** Enlarged estuary cluster. Symbols denote Active, Abandoned, Restored and Reference sites. The park boundary is shown for spatial context. Contains modified Copernicus Sentinel data (2021).

The monitoring network comprised 15 oilfield sites (O01-O15) and six natural reference wetland sites (R01-R06). Oilfield sites extended from 118.7845 to 119.2208°E and from 37.7248 to 37.8716°N. Vegetation was surveyed in June and December 2021 using three quadrats per oilfield site during each survey. The two surveys were analysed separately to distinguish growing-season and winter vegetation conditions. Site-level values, rather than individual quadrats, were the units of comparison. The mean and variation across the six reference wetlands provided a common vegetation benchmark for the oilfield sites.

### 2.2 Operational states and retirement-age classes

Sites were classified by operational status, infrastructure retention and restoration status. Active sites contained operating wells or stations (n = 5). Abandoned sites had ceased production but retained pads or facilities (n = 4). Restored sites had infrastructure removed and ecological restoration implemented (n = 6). Reference sites had no evident oilfield disturbance (n = 6). The term Restored denotes this management state, rather than an assumption that ecological recovery was complete. Retirement age was calculated from production cessation for Abandoned sites and from completion of infrastructure removal for Restored sites, relative to the 2021 sampling year. Restored sites included two <3 year sites, two 3–5 year sites and two 5–10 year sites. Abandoned sites included two 3–5 year sites and two 5–10 year sites. Direct comparisons between retired management states were made within the two shared age classes; Active sites provided an operational baseline and were not assigned a retirement age.

### 2.3 Soil conditions and heavy-metal risk indices

Surface soils were sampled in June and December 2021 using non-metallic tools and transported to the laboratory in sealed polyethylene bags. Samples were air-dried, homogenised and passed through a 2-mm sieve; subsamples for chemical analysis were further passed through a 1-mm sieve. For pH and salinity, 1:5 soil:water extracts were prepared by repeated shaking and filtration. Soil pH was measured potentiometrically and salinity with a salinometer. Aqueous soil extracts are also widely used to characterise soil conditions in Yellow River Delta wetlands (Bai et al., 2014; Yu et al., 2022). Soil chemical measurements included As, Cd, total chromium (Cr), Cu, Pb and Hg. Cd, Cu and Pb were measured by atomic absorption spectrometry, and As and Hg by atomic fluorescence spectrometry.

Metal status was characterised using single-factor pollution indices for As, Cd, Cr, Cu and Pb, the pollution load index (PLI), and the potential ecological risk index (RI). The single-factor index for an element was its measured concentration divided by the corresponding assessment benchmark. The Cd benchmark was 0.20 mg kg^-^¹, so a Cd index of one denotes that concentration. PLI was calculated as the geometric mean of five contamination factors, each defined as the concentration of an element divided by its local reference concentration (Tomlinson et al., 1980). Thus, the Cd single-factor index and the contamination factor used in PLI have different denominators. A PLI of one denotes a geometric mean contamination factor of one across the five elements.

RI was calculated as the sum of element-specific risk scores, each obtained by multiplying the local-reference contamination factor by its toxicity-response coefficient (Hakanson, 1980). The coefficients were 10 for As, 30 for Cd, 2 for Cr, 5 for Cu and 5 for Pb. Cd contributions were expressed both as risk scores and as percentages of total RI. Hg was not included in the five-element PLI and RI calculations. These indices describe concentration-based contamination and potential risk; they do not measure biological toxicity directly. Cd index, PLI and RI were selected for the retirement-stage comparisons to represent single-element contamination, combined enrichment and toxicity-weighted risk, respectively.

Mean soil-metal concentrations at oilfield and reference wetlands are presented in **Table 1** for context. Reference soil conditions are expressed as group means ± SD.

**Table 1:** Mean soil metal concentrations and contrasts with reference wetlands.

Element	Oilfield mean (mg kg^-^¹)	Reference mean (mg kg^-^¹)	Oilfield/reference ratio
As	9.65	12.17	0.79
Cd	0.18	0.18	1.00
Total Cr	49.00	41.93	1.17
Cu	19.30	19.75	0.98
Pb	22.39	18.70	1.20
Hg	ND	ND	—

Oilfield concentrations are means across 15 monitoring sites. Reference concentrations are group means. Oilfield/reference ratios were calculated by dividing the oilfield mean by the corresponding reference mean and are approximate. ND, not detected; —, ratio not estimable because Hg was not detected in either group.

### 2.4 Vegetation variables

Vegetation records included cover, species identity, density, height and frequency. Species richness was the number of species recorded at a site. Relative abundance was calculated as the density of each species divided by the total density of all species at that site. Gini-Simpson diversity was one minus the sum of squared relative abundances, and Shannon diversity was the negative sum of each relative abundance multiplied by its natural logarithm. These measures distinguish the number of species from their distribution of abundance, allowing cover, richness and diversity to be compared separately. Such complementary vegetation attributes have been used to assess restoration development in Yellow River Delta reed wetlands (Wang et al., 2012).

June vegetation was used for retirement-stage comparisons as the growing-season survey, while December provided a seasonal comparison. Seasonal rank-abundance curves were constructed by summing the density of each species across oilfield sites, converting the totals to relative abundance and ordering species from most to least abundant. Species occurrence was expressed as the number of oilfield sites at which each species was recorded. These pooled community summaries describe the seasonal species assemblage, whereas retirement-stage comparisons retain the site as the unit of observation.

### 2.5 Data analysis

The spatial chronosequence compared sites of different retirement ages rather than following individual sites through time (Walker et al., 2010). Oilfield-reference contrasts were summarised separately for each survey using arithmetic means, sample standard deviations and observed ranges. State-by-age comparisons used individual site values and group means. Boxplots show medians and interquartile ranges, with whiskers extending to the most extreme observations within 1.5 interquartile ranges; individual observations are also displayed. Each retired state-by-age group contained two sites, so these comparisons were descriptive and no temporal regression was fitted to the three age classes.

Pearson correlations described covariation among the five metal single-factor index series across the 15 oilfield sites. Spearman rank correlations were used to examine pH, salinity, Cd index and PLI in relation to June cover, richness, Gini-Simpson diversity and Shannon diversity. This analysis used the same 14 sites with complete paired measurements for all combinations. Two-sided P values were reported, with P < 0.05 used to identify associations for display. Straight lines in the pH scatterplots are visual summaries; the reported coefficients and P values are from the rank correlations. All analyses used Python with pandas, NumPy and SciPy, and figures were produced with Matplotlib.

## 3 Results

### 3.1 Oilfield sites differed from reference wetlands in soil conditions and vegetation structure

Oilfield sites had higher mean pH than reference wetlands (9.28 ± 0.31 versus 8.03 ± 0.05; n = 14 oilfield sites) and lower mean salinity (0.46 ± 0.53 versus 1.90 ± 0.52 ppt; **Figures 2i-l**). Oilfield pH ranged from 8.70 to 9.90, with every observed value above the reference mean. Salinity ranged from 0.10 to 2.20 ppt. O06 had the highest salinity, while values at the remaining 13 sites did not exceed 0.70 ppt. Thus, the salinity distribution included one site above the reference mean and a larger group of substantially lower values.

**Figure 2:**
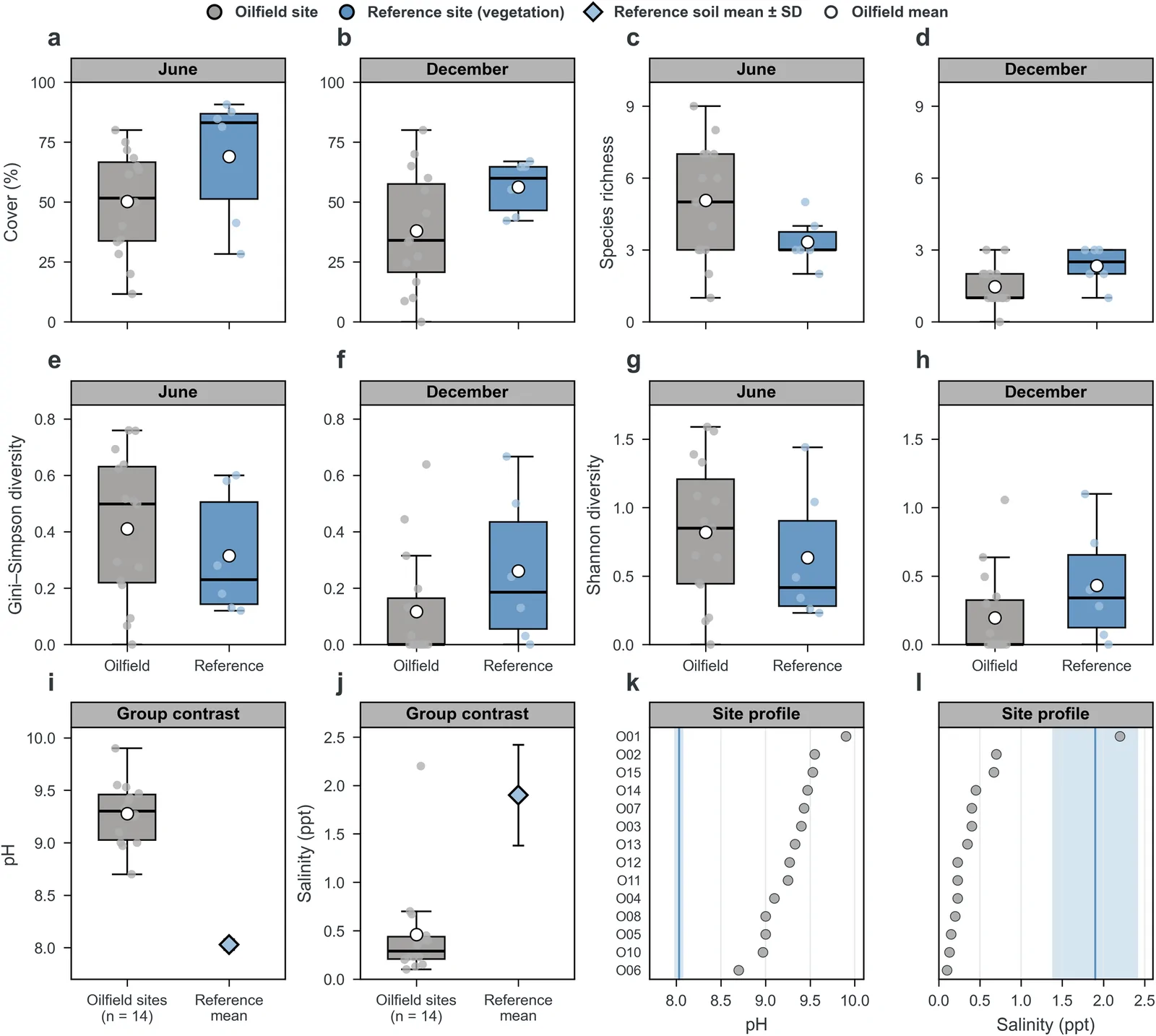
Background contrasts between oilfield sites and natural reference wetlands. **(a–d)** Cover and species richness in June and December. **(e–h)** Gini-Simpson and Shannon diversity in June and December. Vegetation panels (a-h) include 15 oilfield sites and six reference wetlands per survey. **(i, j)** Oilfield and reference summaries for pH and salinity. **(k, l)** Site-level pH and salinity profiles. Filled coloured points show site values; white circles show oilfield means. Soil panels **(i–l)** include 14 oilfield sites; reference soil conditions are shown as reported group means ± SD.

Vegetation cover was lower at oilfield sites in both surveys. Mean June cover was 50.29 ± 21.27% at oilfield sites and 69.00 ± 26.98% at reference sites, a difference of 18.71 percentage points. Mean December cover was 38.00 ± 24.23% and 56.23 ± 11.11%, respectively, a difference of 18.23 percentage points. Oilfield cover ranged from 11.67 to 80.00% in June and from 0 to 80.00% in December. The corresponding reference ranges were 28.30-90.70% and 42.20-67.00% (**Figures 2a, b**).

The direction of the diversity contrasts changed between surveys. In June, mean richness was 5.07 species at oilfield sites and 3.33 at reference sites; mean Gini-Simpson diversity was 0.411 versus 0.315, and Shannon diversity was 0.819 versus 0.633. In December, mean richness was 1.47 species at oilfield sites and 2.33 at reference sites. Mean Gini-Simpson diversity was 0.117 at oilfield sites and 0.261 at reference sites. Mean Shannon diversity was 0.195 at oilfield sites and 0.431 at reference sites. Oilfield richness ranged from one to nine species in June and from zero to three in December (**Figures 2c-h**; **Supplementary Table S2**).

### 3.2 Cd dominated the site-level heavy-metal risk signal

Mean Cr and Pb concentrations at oilfield sites were approximately 17% and 20% higher than the corresponding reference means, whereas mean As was approximately 21% lower. Mean Cd and Cu concentrations were similar to the reference means (**Table 1**). At the site level, single-factor pollution indices across the 15 oilfield sites averaged 0.64 for As, 0.92 for Cd, 0.54 for Cr, 0.55 for Cu and 0.64 for Pb. Cd indices ranged from 0.66 to 1.40. O02, O03 and O10 exceeded one, with values of 1.37, 1.21 and 1.40, respectively. The other four elements remained below one at all sites: their ranges were 0.53-0.90 for As, 0.41-0.75 for Cr, 0.40-0.86 for Cu and 0.48-0.87 for Pb (**Figure 3a**).

**Figure 3:**
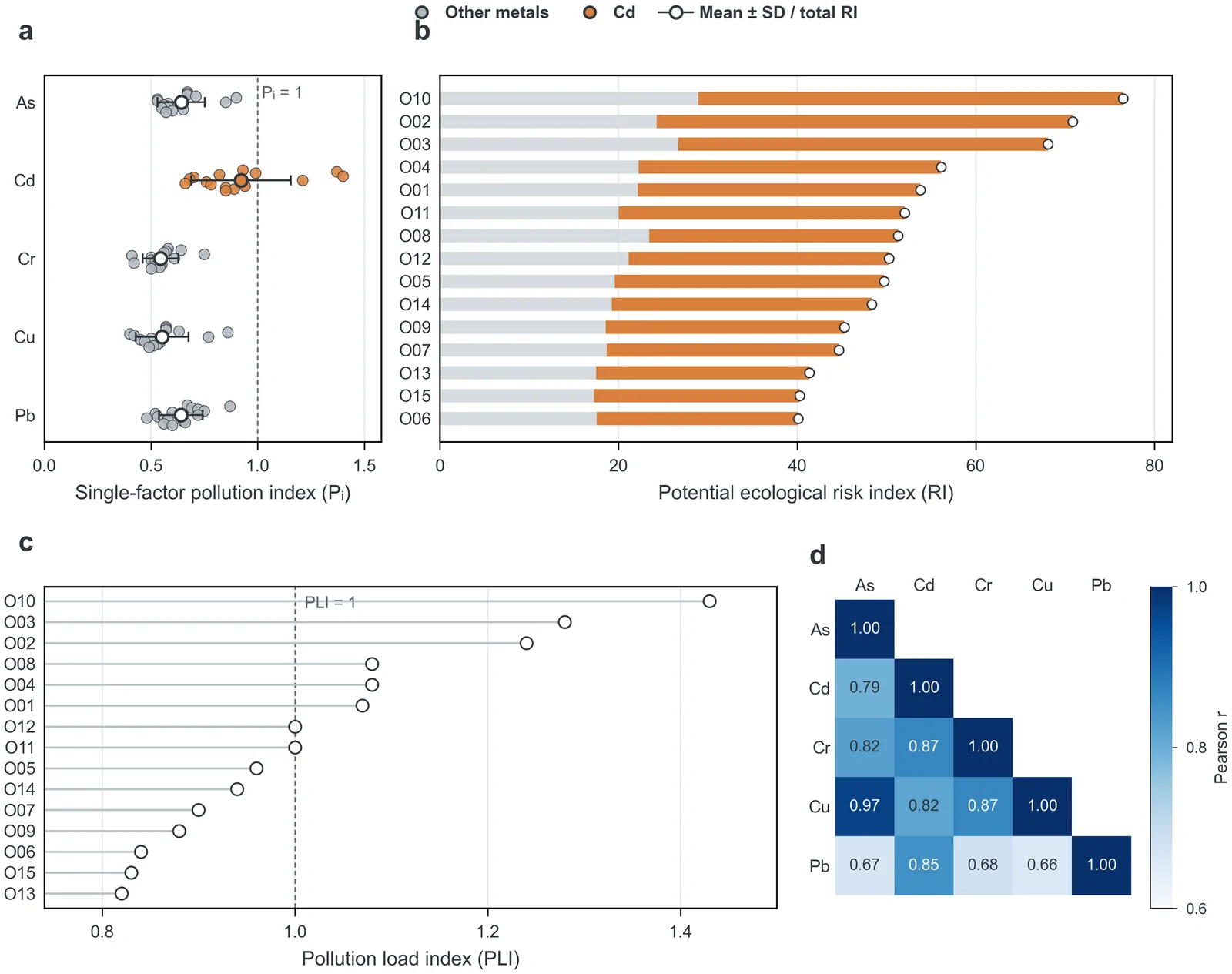
Site-level metal pollution indices and potential ecological risk in oilfield-associated wetlands. **(a)** Single-factor pollution indices for As, Cd, Cr, Cu and Pb. **(b)** Cd and non-Cd contributions to RI. **(c)** Site-level PLI. **(d)** Pearson correlations among the five single-factor index series. Points show site values; white symbols show means or total RI as indicated. Dashed lines in **(a, c)** mark index values of 1. In a, this denotes the element-specific assessment benchmark; in **(c)**, it denotes a geometric mean contamination factor of one relative to local reference concentrations. PLI, pollution load index; RI, potential ecological risk index.

PLI ranged from 0.82 to 1.43 (mean 1.02). Six sites had values above one, comprising all five Active sites and Abandoned site O03; O11 and O12 both had values of 1.00. RI ranged from 40.10 at O06 to 76.49 at O10, with a mean of 52.58. The three largest RI values occurred at O10 (76.49), O02 (70.83) and O03 (68.09), the same sites with Cd indices above one. Cd accounted for 54.3-65.7% of total RI across sites, with an average contribution of 59.4% (**Figures 3b, c**; **Supplementary Table S1**).

All pairwise correlations among metal indices were positive, with Pearson coefficients ranging from 0.658 to 0.972. As and Cu showed the highest correlation (r = 0.972), followed by Cd-Cr (r = 0.867), Cr-Cu (r = 0.866) and Cd-Pb (r = 0.850). These correlations describe the spatial covariation of the index values across oilfield sites (**Figure 3d**).

### 3.3 Metal-risk indices across management states and retirement-age classes

Active sites had a mean Cd index of 1.10, mean PLI of 1.18 and mean RI of 61.70. Each retired state-by-age group had lower means than the Active group for all three indices. Within Active sites, however, Cd index ranged from 0.82 to 1.40 and RI from 51.28 to 76.49, overlapping the values observed at several retired sites (**Figure 4**). Site-level Cd index and RI values are provided in **Supplementary Table S1**.

**Figure 4:**
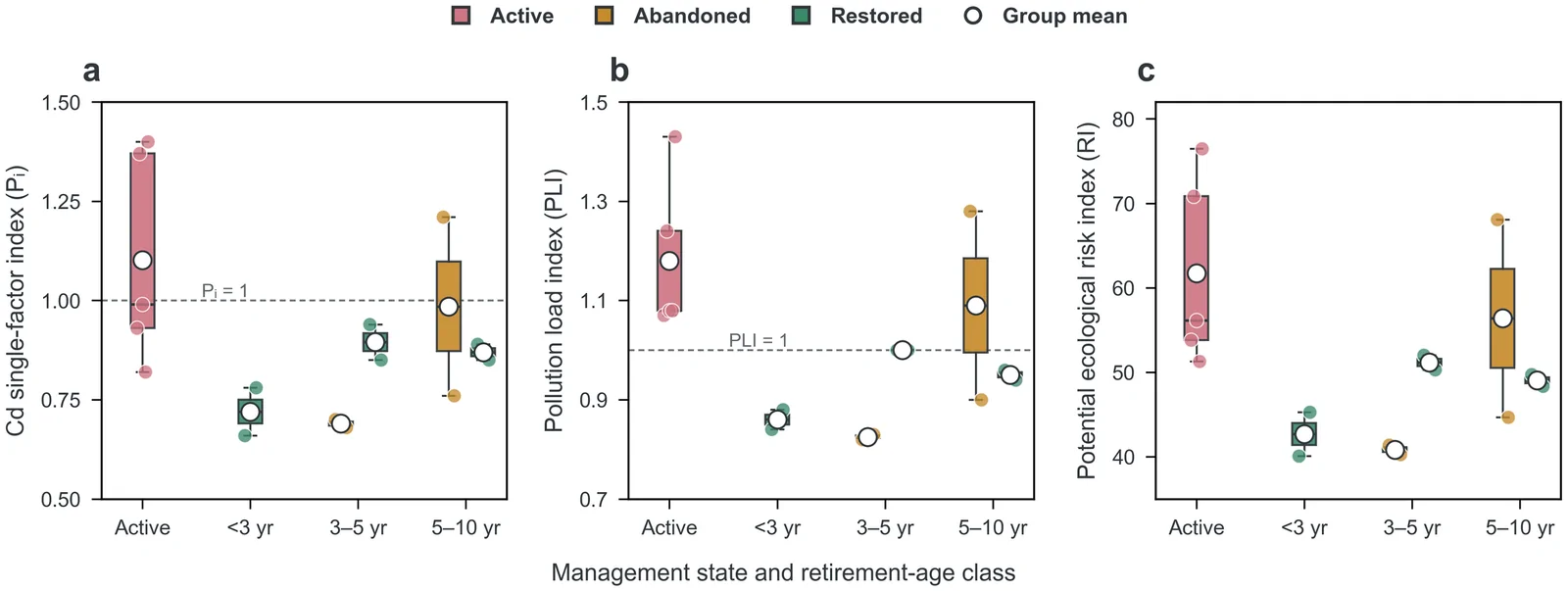
Metal-risk indices by management state and retirement-age class. **(a)** Cd single-factor index. **(b)** PLI. **(c)** RI. Active is an operational baseline rather than a retirement-age class. Retirement ages are relative to the 2021 survey year. At 3–5 and 5–10 years, Abandoned and Restored groups are plotted side by side. Boxes show the median and interquartile range; whiskers extend to the most extreme observations within 1.5 interquartile ranges. Coloured points show individual sites and open circles show arithmetic group means. Active n = 5; each retired state-by-age group n = 2. Dashed lines in a and b mark index values of one, using the respective assessment and local-reference benchmarks.

Among Restored sites, mean Cd index was 0.72 at <3 years, 0.90 at 3–5 years and 0.87 at 5–10 years. Mean PLI was 0.86, 1.00 and 0.95, and mean RI was 42.69, 51.16 and 49.04, respectively. All three indices were lowest in the youngest class, increased in the intermediate class and were slightly lower in the oldest class. No Restored site had a Cd index above one. The three indices therefore showed no monotonic decrease across the Restored retirement-age sequence.

At 3–5 years, Abandoned sites had mean Cd index, PLI and RI values of 0.69, 0.83 and 40.83, respectively; Restored means were higher for all three indices, with RI higher by 10.33. At 5–10 years, the corresponding Abandoned means were 0.99, 1.09 and 56.38, whereas Restored means were lower, with RI lower by 7.34. The two older Abandoned sites differed substantially: O03 had a Cd index of 1.21 and RI of 68.09, whereas O07 had values of 0.76 and 44.67. By comparison, RI at the two oldest Restored sites was 49.74 and 48.34 (**Figure 4**).

### 3.4 Vegetation metrics across management states and retirement-age classes

Mean June cover at Restored sites increased across the three age classes from 15.8% at <3 years to 59.2% at 3–5 years and 64.2% at 5–10 years. The difference between the youngest and oldest classes was 48.3 percentage points. The two youngest sites had cover values of 11.67% and 20.00%, below the lowest reference value of 28.30%. Cover in the two older classes ranged from 50.00 to 68.33%, within the reference range. Relative to the reference mean of 69.00%, the cover differences were 53.2, 9.8 and 4.8 percentage points across the successive Restored age classes (**Figure 5a**).

**Figure 5:**
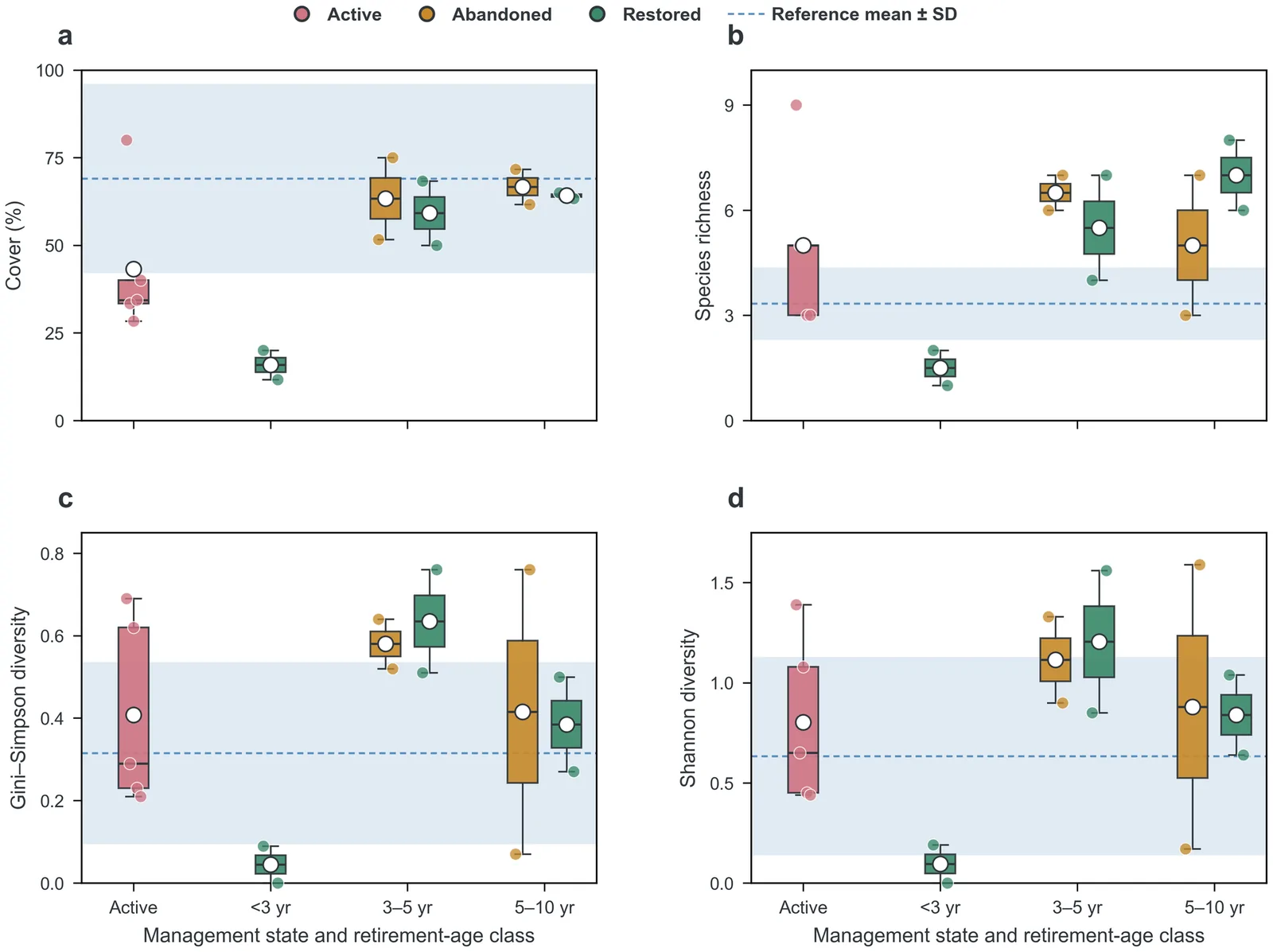
Peak growing-season vegetation responses by management state and retirement-age class. **(a)** Cover. **(b)** Species richness. **(c)** Gini-Simpson diversity. **(d)** Shannon diversity. Shaded blue bands and dashed lines show the mean ± SD and mean, respectively, of six reference wetland vegetation values. Active is an operational baseline. Boxes show medians and interquartile ranges, with whiskers extending to the most extreme observations within 1.5 interquartile ranges. Coloured points show individual sites; open circles show arithmetic group means. Active n = 5; each retired state-by-age group n = 2.

Mean richness at Restored sites was 1.5, 5.5 and 7.0 species across the same sequence. The oldest class contained six and eight species per site, both above the reference range of two to five species. Diversity did not follow this monotonic increase: mean Gini-Simpson diversity was 0.045, 0.635 and 0.385, and mean Shannon diversity was 0.095, 1.205 and 0.840. Both diversity indices peaked at 3–5 years. The youngest class included O09, where one species was recorded and both diversity indices were zero (**Figures 5b–d**).

Abandoned sites had mean cover of 63.3% at 3–5 years and 66.7% at 5–10 years, slightly above the corresponding Restored means of 59.2% and 64.2%. At 3–5 years, Abandoned sites had higher mean richness but lower mean Gini-Simpson and Shannon diversity than Restored sites. At 5–10 years, this ordering was reversed: Abandoned means were 5.0 species, 0.415 and 0.880, compared with Restored means of 7.0 species, 0.385 and 0.840. Active sites had mean cover of 43.2% and richness of 5.0 species, but their cover ranged from 28.33 to 80.00%.

Beyond the retirement-stage comparisons, the pooled species assemblage differed between the June and December surveys (**Supplementary Figure S1**). Twenty-three species were recorded across oilfield sites in June, compared with six in December. *Phragmites australis* occurred at 12 of 15 sites in June and 13 of 15 in December. Its share of summed species density increased from 28.4% to 87.4%. The two most abundant species in both surveys, *Suaeda glauca* and *Phragmites australis*, together accounted for 63.7% of pooled density in June and 98.7% in December, producing a steeper winter rank-abundance distribution.

### 3.5 Soil pH was associated with vegetation cover and richness

Across the 14 oilfield sites with complete paired measurements, pH was positively associated with June cover (Spearman r = 0.548, P = 0.043) and richness (r = 0.561, P = 0.037). The correlations with Gini-Simpson diversity (r = 0.407, P = 0.148) and Shannon diversity (r = 0.365, P = 0.199) were also positive but did not reach P < 0.05. Salinity was negatively correlated with all four vegetation metrics, with coefficients between -0.477 and -0.406. Its largest absolute correlation was with Gini-Simpson diversity (r = -0.477, P = 0.085; **Figure 6a**).

**Figure 6:**
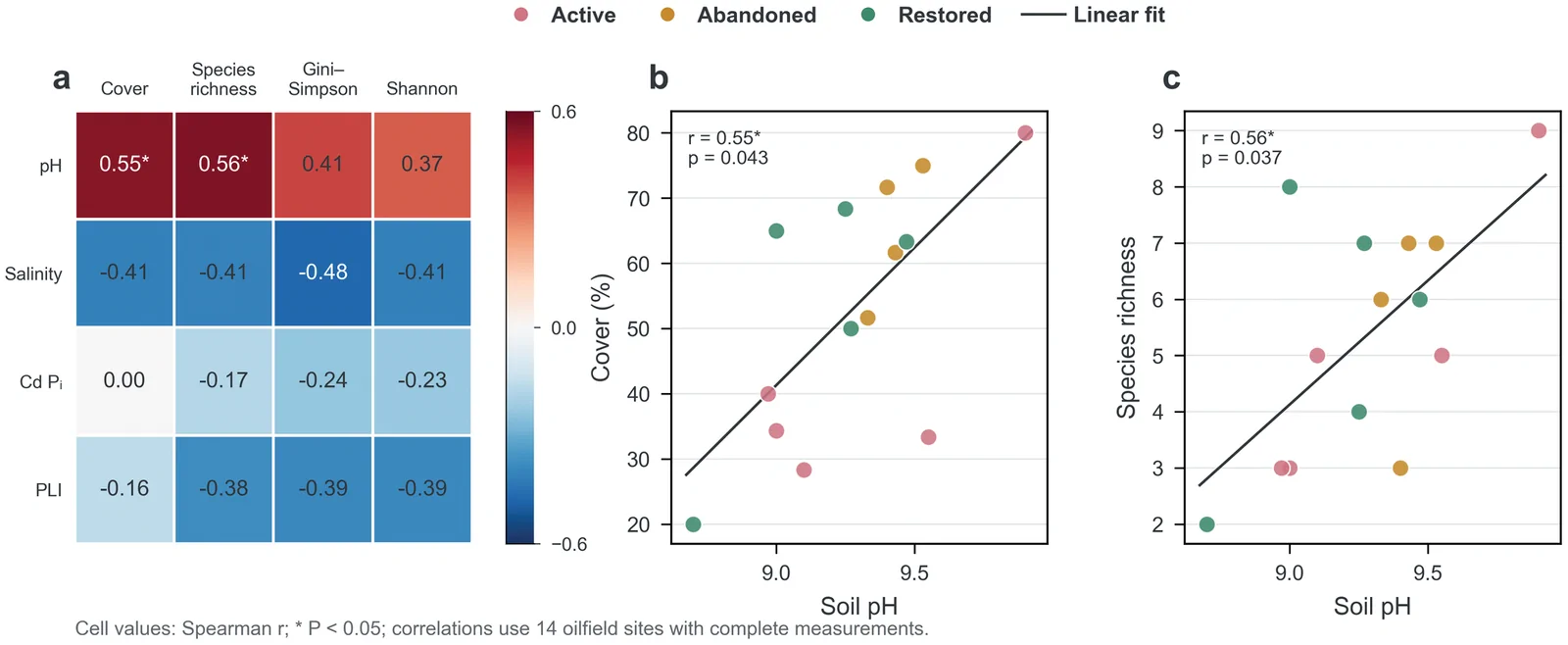
Environmental correlates of vegetation recovery in oilfield-associated wetlands. **(a)** Spearman correlations between soil conditions or pollution-risk indicators and June vegetation responses. **(b, c)** Significant pH associations with cover and species richness. Points are coloured by management state; black lines show linear fits used only as visual summaries. Coefficients and P values are from Spearman rank correlations. Correlations use 14 oilfield sites with complete measurements. Asterisks indicate P < 0.05.

Cd index and PLI showed no significant correlations with the vegetation metrics. The Cd-cover correlation was close to zero (r = 0.004, P = 0.988), while Cd correlations with richness and the two diversity indices ranged from -0.239 to -0.175. PLI correlations were negative, ranging from -0.389 to -0.159, with P values of 0.169-0.588. Of the 16 soil-vegetation correlations examined, only the two pH associations with cover and richness had P < 0.05 (**Figure 6**).

## 4 Discussion

### 4.1 Production cessation and ecological recovery are not equivalent

Production cessation should not be treated as a proxy for wetland recovery. Metal-risk means were lower in every retired group than in Active sites, yet the retired groups did not form a consistent sequence towards progressively lower risk. Vegetation showed a different pattern: cover at older Restored sites approached the reference mean, while richness exceeded it and diversity peaked at an intermediate stage. Taken together, these observations support a component-specific view of recovery rather than a single ranking of sites from degraded to recovered. Such a distinction is consistent with ecological legacies, through which historical disturbance continues to influence ecological organisation after the original pressure has ceased (Cuddington, 2011).

The continued separation of oilfield and reference soil conditions is also relevant. A wetland with relatively low metal-risk indices may still occupy a different hydrological and edaphic setting from the reference wetlands. Oil wells and roads have fragmented the Yellow River Delta landscape and altered connectivity (Bi et al., 2011; Wu et al., 2023), providing plausible pathways for persistent habitat differences. Oil-spill studies similarly show that visible plant recovery can occur without reversal of all physical habitat damage (Silliman et al., 2012). Although the disturbance histories differ, this comparison underscores why vegetation cover, soil condition and contamination should be assessed as complementary outcomes rather than interchangeable signs of recovery.

### 4.2 Metal-risk legacies varied among sites and retirement stages

Cd was the main contributor to potential ecological risk, accounting for more than half of RI at every site. This dominance reflects both its concentration relative to the local reference and its high toxicity-response coefficient in the Hakanson framework (Hakanson, 1980). Regional surveys have likewise highlighted Cd: a Yellow River Delta assessment found relatively low overall contamination but identified Cd as the principal potential threat, and a Bohai Sea wetland survey reported the highest pollution index for Cd (Miao et al., 2020; Lv et al., 2024). The three sites with Cd indices above one were also the three sites with the highest RI. Their coexistence with a regional mean Cd concentration close to the reference mean illustrates how averaging can obscure local contamination. Cd index, PLI and RI nevertheless answer different questions: the first compares Cd with a specified assessment benchmark, PLI integrates multielement enrichment, and RI weights enrichment by potential toxicity (Tomlinson et al., 1980; Hakanson, 1980). Agreement among them at the highest-risk sites is therefore more informative than any index considered alone.

The retirement-age pattern does not support progressive chemical-risk attenuation over the sampled interval. The youngest Restored group had the lowest mean indices, and the contrast between Restored and Abandoned sites changed direction between 3–5 and 5–10 years. The high RI at O03 contributed to the older Abandoned mean, whereas the two oldest Restored sites had similar RI values. These observations point to strong site dependence. Regional studies have related metal distributions to vegetation type, sediment texture and seasonal water-sediment dynamics (Bai et al., 2014; Yu et al., 2022). Soil organic matter and texture also help explain metal accumulation across freshwater wetlands and salt marshes in the Yellow River Estuary (Zhao et al., 2021). In addition, changes in pH, salinity and redox conditions can redistribute metals between solid and dissolved phases without eliminating them from the wetland (Du Laing et al., 2009). These processes are plausible explanations for spatially variable trajectories, rather than mechanisms demonstrated by the present chronosequence.

The positive correlations among metal indices indicate spatial covariation but do not identify a common pollution source. Shared sediment properties, regional inputs and local disturbance may each contribute. Long-term effects of residual hydrocarbons on reed communities have also been documented in the Yellow River Delta (Zhu et al., 2015), emphasising that metal indices represent only one part of the chemical legacy of petroleum development. Site-specific assessment remains necessary even where most index values are below their comparison benchmarks.

### 4.3 Vegetation recovery involved reorganisation rather than uniform return to reference means

The cover contrast among Restored age classes is consistent with plant establishment following initial disturbance: the two youngest sites were sparsely vegetated, whereas cover in both older groups was within the observed reference range. However, increasing richness is not necessarily equivalent to convergence towards a natural wetland. Both oldest Restored sites were richer in species than any of the six reference sites, while diversity was lower than in the intermediate Restored class. A comparable pattern has been observed in restored reed wetlands of the Yellow River Delta, where cover approached natural values but richness and diversity first increased and subsequently declined (Wang et al., 2012). Species replacement and changing dominance can therefore accompany recovery without producing a monotonic rise in every diversity measure.

Structural recovery refers here to the return of vegetation structure, such as cover, whereas compositional recovery concerns the re-establishment of a reference species assemblage. Managed-realignment marshes can attain species richness comparable to natural marshes while retaining different species assemblages (Mossman et al., 2012). Following oil exposure, individual marsh species can also recover at different rates, altering community dominance even as aboveground vegetation returns (Lin and Mendelssohn, 2012; Lin et al., 2016). A synthesis of Deepwater Horizon studies further found that belowground biomass could remain impaired while several aboveground attributes showed recovery (Zengel et al., 2022). Our seasonal observations, including the strong December dominance of *Phragmites australis*, show that richness and abundance structure depend on survey season as well as management state. Cover recovery should therefore be interpreted as one aspect of vegetation development, not as proof that the original community or its ecological functions have been fully re-established. This interpretation accords with broader evidence for persistent structural and functional recovery deficits in restored wetlands (Moreno-Mateos et al., 2012, Moreno-Mateos et al., 2017).

### 4.4 Soil conditions and metal-risk indices showed contrasting associations with vegetation

Vegetation and metal-risk indices were not aligned across the Restored chronosequence. The youngest group combined the lowest vegetation cover and richness with the lowest metal-risk means, while older groups had greater vegetation development without correspondingly lower chemical indices. Likewise, Cd index and PLI were not significantly correlated with the June vegetation metrics. The data therefore do not support a simple sequence in which declining metal indices alone explain revegetation. This does not imply that contamination is ecologically irrelevant: concentration-based indices do not directly represent metal bioavailability, which varies with sediment chemistry and hydrological conditions (Du Laing et al., 2009).

The positive pH associations apply to variation within the oilfield sites; they do not mean that increasing pH would restore reference conditions. Indeed, reference wetlands had lower mean pH and higher mean cover. Soil pH may therefore covary with other habitat attributes within the disturbed landscape. Restoration studies in the delta have linked vegetation development to water depth and salinity (Cui et al., 2009; Wang et al., 2012), while saline habitat heterogeneity also structures local reed populations (Gao et al., 2012). Experiments in coastal salt marshes have shown that flooding and salinity can interact with competition to determine plant zonation (Pennings et al., 2005). Elevation and sediment redox conditions provide additional controls on salt-marsh colonisation (Davy et al., 2011). The correlation results consequently identify habitat-related hypotheses for further testing, not a prescription to alter pH independently of the water and salt regime.

### 4.5 Limitations and management implications

The chronosequence resolves contrasts among retirement stages but cannot separate elapsed time from differences in prior disturbance or habitat setting. With two sites in each retired state-by-age group, the observations do not provide estimates of annual recovery rates or the causal effect of infrastructure removal. Spatial chronosequences are most informative when their environmental comparability is considered alongside age (Johnson and Miyanishi, 2008; Walker et al., 2010). Here, the common reference benchmark describes the ecological target, while the variation among oilfield sites cautions against treating every site as a point on one temporal trajectory. The environmental correlations are exploratory and require independent testing.

The management implication is to distinguish completion of retirement works from attainment of ecological recovery objectives. Lower risk means in the retired groups are encouraging, but the absence of a consistent Restored advantage at comparable ages means that removal alone should not be assumed to resolve all ecological constraints. Restoration actions are more usefully matched to demonstrated local barriers than prescribed solely from site age (Jones et al., 2018). For contamination assessment, local Cd hotspots merit continued attention. For vegetation and habitat assessment, revegetation should be evaluated using cover, diversity and characteristic species alongside soil conditions. This combined assessment is consistent with restoration frameworks that evaluate multiple ecological attributes against reference conditions and explicit recovery objectives (Wortley et al., 2013; Gann et al., 2019). Repeated observations spanning cessation, infrastructure removal and subsequent vegetation development would test whether the differences observed here represent persistent trajectories and whether particular interventions improve them.

## 5 Conclusions

Oil-well retirement in the Yellow River Delta was associated with contrasting metal-risk and vegetation patterns. Retired groups had lower mean metal-risk indices than Active sites, but these indices did not decline monotonically across retirement ages; Cd remained the dominant contributor to potential ecological risk. Older Restored sites had cover closer to the reference mean, while richness and diversity followed different patterns. Comparisons between Abandoned and Restored sites also varied with age and indicator. Recovery assessment should therefore combine site-specific contamination evaluation with vegetation and habitat assessment. Neither elapsed time nor infrastructure removal alone provides sufficient evidence of ecological recovery.

## Data Availability

The original contributions presented in the study are included in the article/**Supplementary Material**. Further inquiries can be directed to the corresponding author.
