# Supplementary material for: Ecological recovery trajectories of coastal wetlands following oil-well abandonment in the Yellow River Delta
Source: Front Plant Sci. 2026 Oct 22;17:1989517. doi: 10.3389/fpls.2026.1989517 (PMC13736748; doi:10.3389/fpls.2026.1989517)
Supplement: Supplementary file 1 [file Table1.docx]

# Supplementary material

## Supplementary Figure S1

Supplementary Figure S1. Seasonal community structure across oilfield-associated sites.

a, Rank-abundance distributions in June and December 2021. Relative abundance is the summed density of a species across oilfield sites divided by the summed density of all species in that survey. b, Species occurrence across the 15 oilfield sites. Each seasonal curve ranks species independently from most to least abundant.

## Supplementary Table S1

Supplementary Table S1. Site-level Cd contamination and potential ecological risk indices.

| **Site** | **Cd index** | **Cd risk score** | **RI** |
| --- | --- | --- | --- |
| O01 | 0.93 | 31.63 | 53.79 |
| O02 | 1.37 | 46.55 | 70.83 |
| O03 | 1.21 | 41.42 | 68.09 |
| O04 | 0.99 | 33.86 | 56.12 |
| O05 | 0.89 | 30.17 | 49.74 |
| O06 | 0.66 | 22.55 | 40.10 |
| O07 | 0.76 | 26.01 | 44.67 |
| O08 | 0.82 | 27.85 | 51.28 |
| O09 | 0.78 | 26.71 | 45.28 |
| O10 | 1.40 | 47.56 | 76.49 |
| O11 | 0.94 | 32.03 | 52.04 |
| O12 | 0.85 | 29.14 | 50.28 |
| O13 | 0.70 | 23.88 | 41.38 |
| O14 | 0.85 | 29.11 | 48.34 |
| O15 | 0.68 | 23.02 | 40.28 |

The Cd index uses an assessment benchmark of 0.20 mg/kg. Cd risk scores use local reference concentrations and a toxicity-response coefficient of 30. RI is the sum of As, Cd, Cr, Cu and Pb risk scores.

## Supplementary Table S2

Supplementary Table S2. Vegetation summaries for oilfield-associated and reference wetland sites in 2021.

| **Indicator** | **June oilfield** | **June reference** | **December oilfield** | **December reference** |
| --- | --- | --- | --- | --- |
| Cover (%) | 50.29 | 69.00 | 38.00 | 56.23 |
| Richness (species) | 5.07 | 3.33 | 1.47 | 2.33 |
| Gini-Simpson diversity | 0.411 | 0.315 | 0.117 | 0.261 |
| Shannon diversity | 0.819 | 0.633 | 0.195 | 0.431 |

Cover, richness and diversity summarise 15 oilfield and six reference wetland sites.
